# An Unexpected Deuterium-Induced Metabolic Switch in Doxophylline

**DOI:** 10.1021/acsmedchemlett.2c00166

**Published:** 2022-07-14

**Authors:** Silvio Aprile, Giorgia Colombo, Marta Serafini, Rosanna Di Paola, Federica Pisati, Irene Preet Bhela, Salvatore Cuzzocrea, Giorgio Grosa, Tracey Pirali

**Affiliations:** † Department of Pharmaceutical Sciences, Università del Piemonte Orientale, 28100 Novara, Italy; ‡ Department of Chemical, Biological, Pharmaceutical and Environmental Sciences, Università di Messina, 98166 Messina, Italy; § Histopathology Unit, Cogentech S.C.a.R.L., 20139 Milan, Italy

**Keywords:** Deuterium, deuterium kinetic isotope effect, metabolic switch, doxophylline

## Abstract

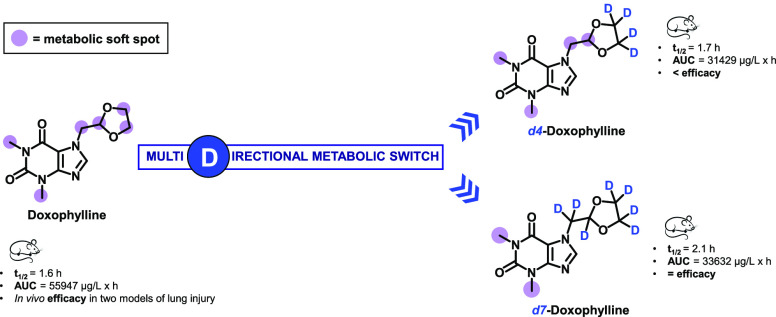

Precision deuteration has become part of the medicinal chemist’s toolbox, but its usefulness can be undermined by unpredictable metabolic switch effects. Herein we report the deuteration of doxophylline, a drug used in the treatment of asthma and COPD that undergoes extensive oxidative metabolism. Labeling of the main metabolic soft spots triggered an unexpected multidirectional metabolic switch that, while not improving the pharmacokinetic parameters, changed the metabolic scenario and, in turn, the pharmacodynamic features in two murine models of lung injury.

Asthma, chronic obstructive pulmonary disease (COPD), and bronchiectasis fall under the umbrella of chronic respiratory diseases and are three closely linked disorders whose phenotype and etiology frequently overlap. Despite the fact that novel medications have emerged, methylxanthines are still indicated as add-on agents for the treatment of both asthma and COPD by GINA^1^ and GOLD^2^ guidelines (*i.e.*, global clinical guidelines for the treatment of asthma and COPD, respectively). In contrast, their use in bronchiectasis is off-label, and their potential in this clinical setting is still elusive and requires further investigations.

Theophylline (**1**) is the most widely used methylxanthine because of its marked bronchodilator activity and anti-inflammatory properties (Figure 1).^3,4^ The precise mechanism of action has not been fully elucidated yet, but its activity spans from nonselective inhibition of phosphodiesterases (PDEs) to the inhibition of phosphoinositide 3-kinases δ (PI3Kδ) and from adenosine receptor antagonism to the restoration of histone deacetylase (HDAC) activity.^5^ Moreover, theophylline significantly reduces the number of neutrophils and eosinophils in the airways. While its use is encouraged by its low cost and high oral bioavailability, theophylline suffers from side effects such as CNS stimulation, cardiac arrhythmias, and gastrointestinal effects, leading to a narrow therapeutic window that warrants strict monitoring of its levels in the blood. It also interferes with CYP1A2, CYP2E21, and CYP3A4, resulting in drug–drug interactions with many drugs metabolized by these pathways.^6^ Because of these drawbacks, theophylline is relegated to second- or third-line therapy in most treatment guidelines.

**Figure 1:**
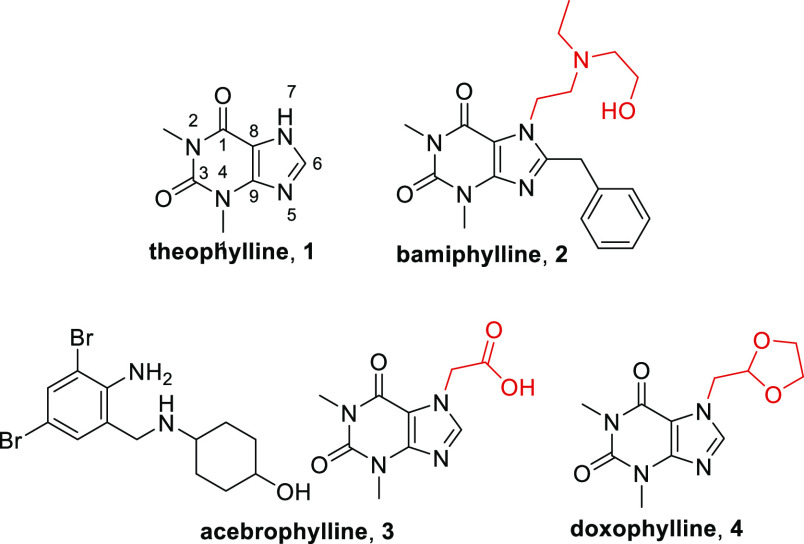
Structures of theophylline and novophyllines.

The clinical benefit of theophylline has spurred in the previous century the development of synthetic analogues named novophyllines with the aim of improving the risk-to-benefit ratio.^7^ These methylxanthines include bamiphylline (**2**), acebrophylline (**3**), and doxophylline (**4**) (Figure 1) and have received regulatory approval for their use in treatment of asthma and COPD in specific parts of the world.

Doxophylline differs from theophylline in that it contains a methylene 1,3-dioxolane group at position 7 (Figure 1), resulting in a different profile.^8^ At the pharmacological level, it has been demonstrated to positively interact with β_2_ adrenoreceptors, with less affinity for α_1_ and α_2_ receptors, eliciting relaxation of blood vessel and bronchial smooth muscles.^9^ Unlike theophylline, it has a low affinity for adenosine receptors, does not inhibit any of the known PDE isoforms (except for PDE_2A1_), and does not interact with HDACs.^10^ With regard to its anti-inflammatory activity, doxophylline has been shown to reduce leukocyte count and recruitment into the airways.^11^ A recent report showed that it reduces the oxidative burst in human monocytes, an effect mediated by the inhibition of protein kinase C (PKC) activity, differently from theophylline.^12^ From a toxicological perspective, indirect comparisons through meta-analyses suggest that it might have a favorable risk-to-benefit ratio compared with theophylline,^13−17^ pointing to this novophylline as a safer therapeutic option for the treatment of chronic respiratory diseases.^18,19^

In humans, doxophylline is readily absorbed after oral administration but promptly eliminated, requiring multiple daily dosing to ensure efficient plasma levels.^8^*In vitro* metabolic studies in rat liver microsomes identified theophylline and the 7-hydroxyethyl ester of 7-theophylline acetic acid (T-COOH, **6**) as metabolites of doxophylline.^20^ Zhao *et al.* showed that in men, doxophylline undergoes a more extensive oxidative metabolism.^21^ After both incubations in human liver fractions and intravenous administration, a metabolic scenario is proposed where different metabolic pathways occur (Figure 2). The main route consists of extensive oxidation of the ethylene moiety on the 1,3-dioxolane ring leading to the formation of 7-theophylline acetaldehyde (T-CHO, **5**), which is further converted to T-COOH **6** or reduced to 7-hydroxyethyltheophylline (etophylline, **7**) (route 1). The second pathway is involved oxidation of the tertiary carbon atom on the 1,3-dioxolane ring, with the formation of the 7-hydroxyethyl ester of T-COOH, which can hydrolyze *in vivo* to afford T-COOH **6** (route 2). In addition, four minor biotransformations are identified (routes 3–6), leading to the formation of theophylline, N-demethylation to form dm-doxophylline (**9**), dehydrogenation of the 1,3-dioxolane ring to give dh-doxophylline (**10**), and oxidation of the xanthine nucleus to give ox-doxophylline (**11**), respectively (Figure 2).

**Figure 2:**
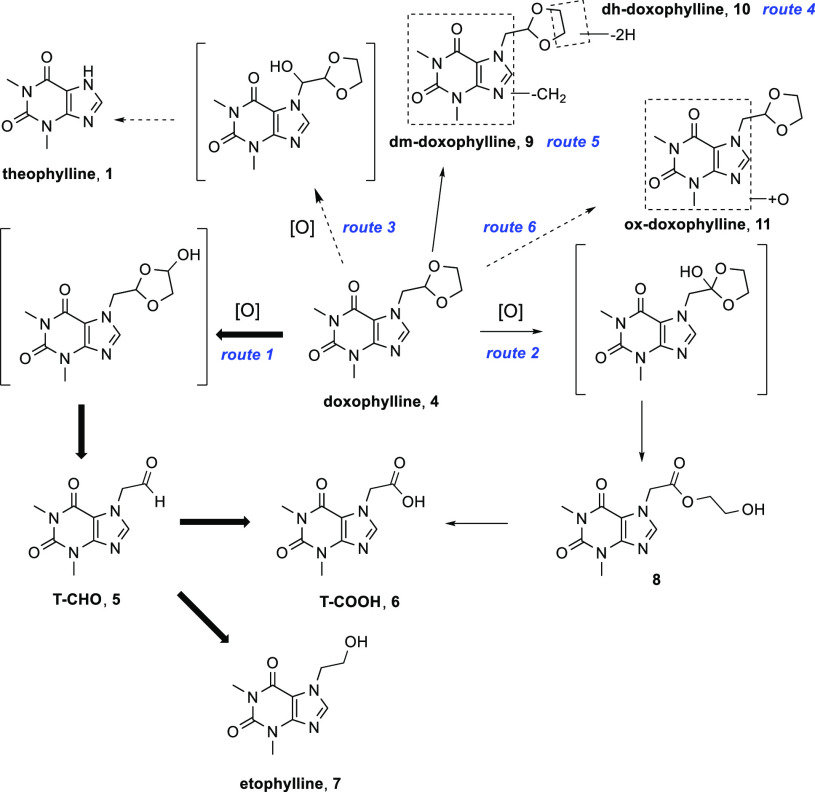
Metabolism of doxophylline in the human liver. Bold arrows represent metabolic conversion extents of >20%. Solid arrows represent metabolic conversion extents of 0.1–20%. Dashed arrows represent metabolic conversion extents of <0.1%.^21^

By virtue of the kinetic isotope effect (KIE), deuterium has been shown to increase the stability toward the C–D cleavage step and induce resistance from oxidative metabolism. Many successful examples have recently been reported in which deuterium incorporation in place of protium leads to a beneficial effect in terms of longer half-life, higher exposure, or reduced toxicity, drug–drug interactions, and interpatient variability.^22−24^ The utility of deuterium labeling in drug R&D is exemplified by the approval in 2017 of deutetrabenazine, the first and to date only approved deuterated drug on the market.^25^ In the field of methylxanthines, a very recent report shows that *d*_9_-caffeine, bearing three deuterated methyl groups, exhibits prolonged systemic and brain exposure compared with its proteo counterpart following oral administration.^26^

With the aim of investigating the effect of deuterium incorporation at the main metabolic soft spots of doxophylline, we synthesized two deuterated analogues, *d*_4_-doxophylline (**16**) and *d*_7_-doxophylline (**20**) (Scheme 1),^27^ and evaluated their pharmacokinetic profiles. In *d*_4_-doxophylline, the ethylene bridge of the 1,3-dioxolane ring was isotopically labeled, while in *d*_7_-doxophylline the entire methylene 1,3-dioxolane group was isotopically labeled. In parallel, we assessed the efficacies of doxophylline, *d*_4_-doxophylline, and *d*_7_-doxophylline in two murine models that resemble chronic lung diseases, where pulmonary injury is induced by bleomycin (BLM) or *Pseudomonas aeruginosa*.

**Scheme 1:**
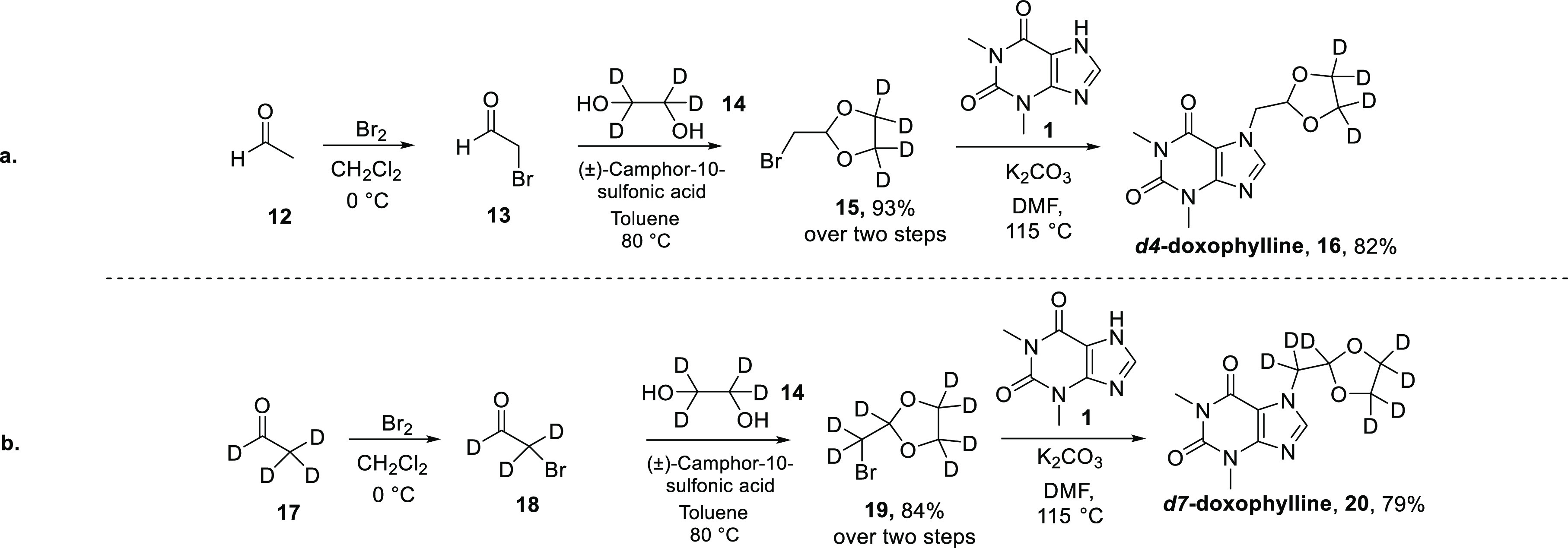
Synthesis of Deuterated Analogues of Doxophylline

To this aim, a straightforward “deuterated pool strategy” was undertaken using commercially available deuterated substrates, namely, *d*_4_-acetaldehyde or/and *d*_4_-ethylene glycol. The general procedure, independent of the isotopic composition, consists of three steps (Scheme 1). Acetaldehyde is brominated using bromine in dichloromethane at 0 °C to give 2-bromoacetaldehyde, which is used in the next step without purification. The addition of ethylene glycol in the presence of (±)-camphor-10-sulfonic acid as the catalyst in toluene at 80 °C affords the corresponding acetal. After purification, the bromomethyl-1,3-dioxolane undergoes nucleophilic substitution with 1,3-dimethyl-1*H*-purine-2,6(3*H*,7*H*)-dione in the presence of potassium carbonate in dimethylformamide at 115 °C to yield the final product. The synthesis proceeded smoothly in high yields for both products and allowed the preparation of the two final compounds on a scale of 15 g.

A comparative pharmacokinetic study was then performed by evaluating the plasma concentration profiles of doxophylline and its deuterated analogues after intravenous and oral single-dose administration in mice and minipigs. Contrary to the expectations, deuteration did not result in increased exposure in either animal species (Tables 1 and 2 and Figure 3). In mice the area under the curve (AUC) decreased for deuterated doxophyllines, especially after oral administration (44% and 40% for *d*_4_-doxophylline and *d*_7_-doxophylline, respectively; Table 1 and Figure 3b), suggesting a significant role of first-pass metabolism. Since the deuterium kinetic isotope effect is often affected by interspecies variability,^28^ a PK study was also performed in minipigs. However, following both oral and intravenous administration, the two deuterated analogues showed very similar pharmacokinetic profiles (Table 2 and Figure 3c,d) compared to doxophylline.

**Table 1:** Pharmacokinetic Data for Doxophylline and Its Deuterated Analogues *d*_4_-Doxophylline and *d*_7_-Doxophylline Following Single-Dose Administration in Mice

parameter	doxophylline	*d*_4_-doxophylline	*d*_7_-doxophylline
Intravenous (20 mg/kg)			
*t*_1/2_ (h)	1.1	1.5	1.0
*C*_max_ (μg/L)	15506	12288	10687
AUC_0–*t*_ (μg L^–1^ h^–1^)	6732	5413	4922
CL (L h^–1^ kg^–1^)	2.9	3.7	4.1
Oral (80 mg/kg)			
*t*_1/2_ (h)	1.6	1.7	2.1
*C*_max_ (μg/L)	18693	16782	19203
AUC_0–*t*_ (μg L^–1^ h^–1^)	55947	31429	33632
CL/F (L h^–1^ kg^–1^)	1.4	2.5	2.4

**Table 2:** Pharmacokinetic Data for Doxophylline and Its Deuterated Analogues *d*_4_-Doxophylline and *d*_7_-Doxophylline Following Single-Dose Administration in minipigs

parameter	doxophylline	*d*_4_-doxophylline	*d*_7_-doxophylline
Intravenous (5 mg/kg)			
*t*_1/2_ (h)	6.2	6.2	6.5
*C*_max_ (μg/L)	7501	7421	8265
AUC_0–*t*_ (μg L^–1^ h^–1^)	47729	49190	47009
CL (L h^–1^ kg^–1^)	0.1	0.1	0.1
Oral (20 mg/kg)			
*t*_1/2_ (h)	11.0	10.8	12.6
*C*_max_ (μg/L)	13961	15811	12998
AUC_0–*t*_ (μg L^–1^ h^–1^)	217271	235790	222429
CL/F (L h^–1^ kg^–1^)	0.07	0.07	0.07

**Figure 3:**
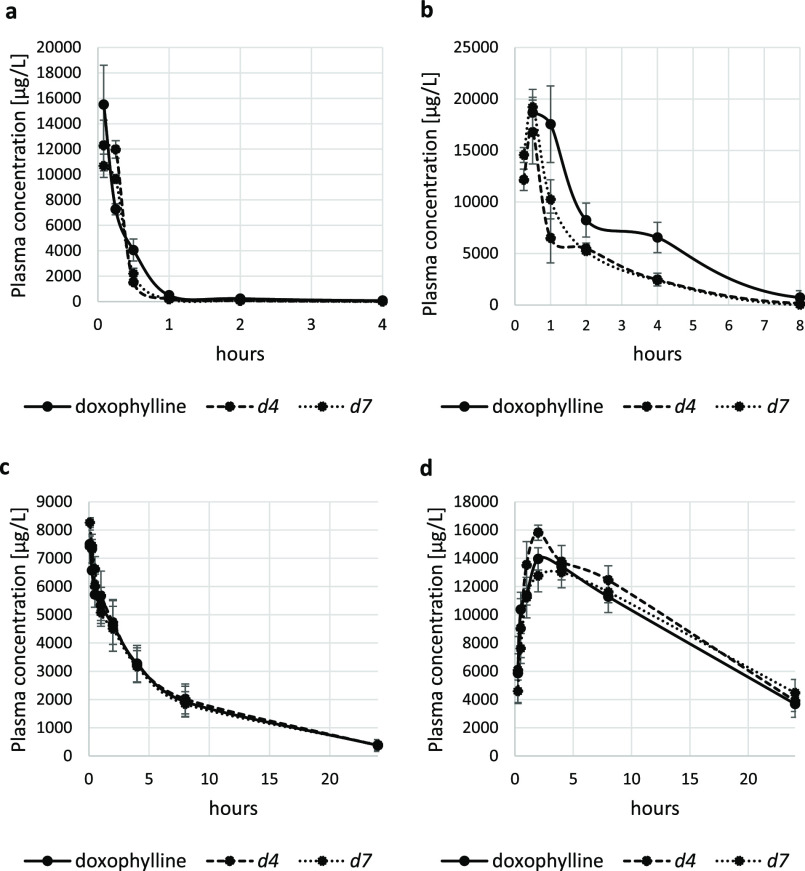
Drug concentration–time curves of doxophylline and its deuterated analogues *d*_4_-doxophylline and *d*_7_-doxophylline after single-dose administration: (a) mice, iv, 20 mg/kg; (b) mice, oral, 80 mg/kg; (c) minipigs, iv, 5 mg/kg; (d) minipigs, oral, 20 mg/kg.

From an analysis of the levels of metabolites in mice plasma, it was seen that deuteration of the dioxolane ring (*d*_4_-doxophylline) triggered considerable alterations in the relative abundances of the metabolites generated. In more detail, increased formation of T-COOH **6** was evident (about 3-fold higher level at 0.5 h), concomitant with a decreased level of etophylline **7** (Figure 4). This is mainly related to a marked increase of the metabolic route 2, in which the electronic influence of the two oxygen atoms adjacent to the target sp^3^ carbon atom greatly facilitates oxene attack and homolytic cleavage of the C–H bond.^29^ Furthermore, a significant increase in the plasmatic levels of theophylline **1** and dm-doxophylline **9** as well as a decrease in the dehydrogenated metabolite dh-doxophylline **10** also occurred (Figure 4). Taken as a whole, these findings suggest that deuteration of the dioxolane ring of doxophylline promotes a multidirectional metabolic switch (Figure 5) that does not result in the desired improvement in the pharmacokinetic parameters.

**Figure 4:**
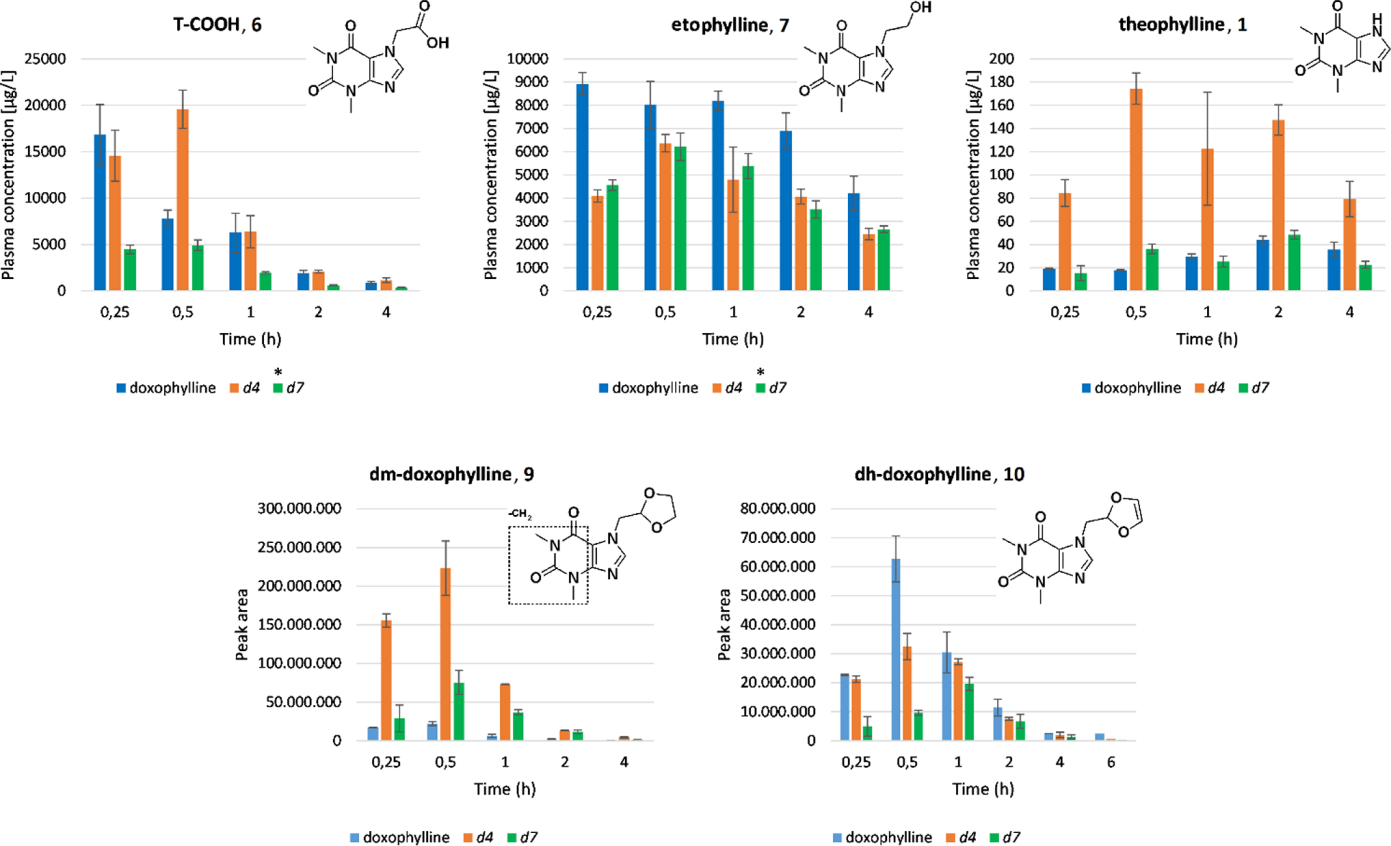
Levels of doxophylline metabolites in mice plasma. *Metabolites were quantified on the basis of a T-COOH calibration curve or nondeuterated metabolite standards.

**Figure 5:**
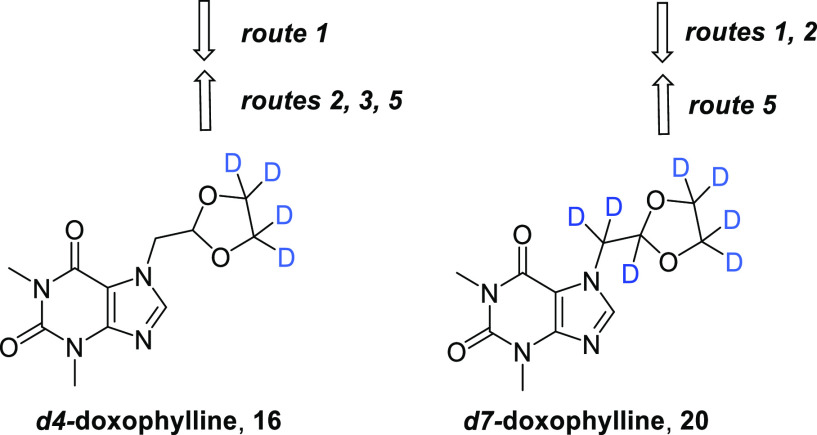
Metabolic switch of the deuterated doxophyllines.

With regard to *d*_7_-doxophylline, only a semiquantitative determination was feasible because its metabolites (with the exception of theophylline) retain deuterium atoms (see the Supporting Information for structures). Indeed, the presence of deuterium lowers the signals in the electrospray ionization source, and the corresponding deuterated standards of the metabolites would have been necessary to perform an accurate quantification. Moreover, no reference standard was available for the quantification of the metabolites dm-doxophylline **9** and dh-doxophylline **10**, and their relative abundances were expressed as peak areas of the corresponding accurate-mass ions. Despite these limitations, we could detect diminished levels of all of the above-mentioned metabolites except for dm-doxophylline **9**, suggesting that, although to a lesser extent than for *d*_4_-doxophylline, the metabolic switch occurs also for *d*_7_-doxophylline and contributes to the reduction of its bioavailability (Figures 4 and 5).

We next investigated whether the different metabolic scenarios of the three compounds would in turn affect their pharmacodynamics because of the possible influence of each metabolite on the *in vivo* efficacy. For instance, T-COOH **6** has been reported to be bronchodilator because of the specific inhibition of PDE-III and IV isozymes,^30^ while etophylline **7** is significantly less active than doxophylline in terms of antibronchospastic and antiasthmatic effects.^31^ To this aim, we exploited two models of lung injury, one induced by BLM and the other by*P. aeruginosa*.

With regard to the BLM-induced lung injury, we treated mice orally with 80 mg/kg doxophylline, *d*_4_-doxophylline, or *d*_7_-doxophylline daily (Figure 6a). As shown in Figure 6b, intratracheal instillation of BLM resulted in marked accumulation of immune cells in bronchoalveolar lavage (BAL) that was significantly reverted by treatment with doxophylline and *d*_7_-doxophylline, while the effect of *d*_4_-doxophylline was of lesser extent and lacked statistical significance. This was also confirmed by the ratio between wet and dry lung (Figure 6c), which reflected decreased liquid accumulation in doxophylline- and *d*_7_-treated mice, and also by determination of MPO, a marker of neutrophil activation, whose activity was decreased in doxophylline- and *d*_7_-treated mice (Figure 6d). Histological examination by hematoxylin and eosin (H&E) staining revealed a decrease in the inflammatory interstitial infiltrate in the groups treated with doxophylline and its analogues, especially in doxophylline- and *d*_7_-treated mice compared with *d*_4_-treated ones (Figure 6e). Finally, to investigate the fibrosis, we performed a semiquantitative analysis on the Masson’s Trichrome- and Picrosirius red-stained sections in order to evaluate the presence and the packing of collagen fibers. A slight tendency toward reduction in thickness and packing of collagen fibers was seen in doxophylline-, *d*_4_-, and *d*_7_-treated mice compared with the vehicle group, and this was mainly observed in the doxophylline and *d*_7_-doxophylline groups, as shown in Figure 6f,g. Furthermore, an interesting association of the inflammatory infiltrate and stromal remodeling was observed. Indeed, the decrease in the interstitial inflammatory state led to a reduction of fibrosis in the doxophylline- and *d*_7_-treated groups, as can be observed in the respective representative images.

**Figure 6:**
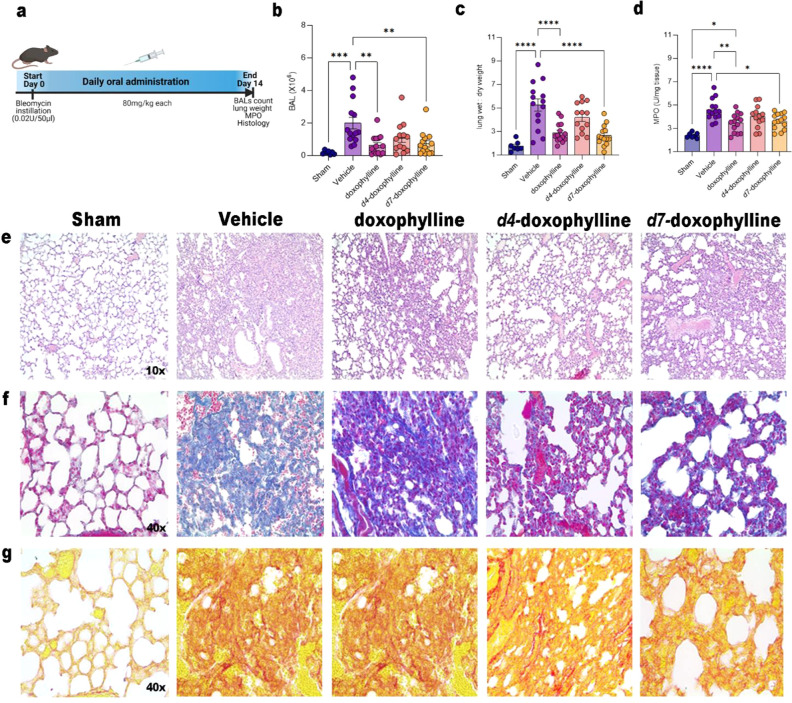
Model of pulmonary fibrosis induced by bleomycin. Doxophylline and *d*_7_-doxophylline attenuate BLM-induced structural damage and lung fibrosis in mice. (a) Representative scheme of the BLM-induced lung injury model. (b) Total BAL cellularity of sham mice (not treated) and bleomycin-treated mice (treated or not with 80 mg/kg doxophylline, *d*_4_-doxophylline, or *d*_7_-doxophylline). Results are reported as mean ± SEM of three independent experiments. (c) Wet/dry lung weight ratio of sham and bleomycin-treated mice (treated or not with 80 mg/kg doxophylline, *d*_4_-doxophylline, or *d*_7_-doxophylline). Results are reported as mean ± SEM of three independent experiments. (d) MPO activity in lungs of sham and bleomycin-treated mice (treated or not with 80 mg/kg doxophylline, *d*_4_-doxophylline and *d*_7_-doxophylline). Results are reported as mean ± SEM of three independent experiments. (e) Representative images of H&E staining of sham and bleomycin-treated mice (treated or not with 80 mg/kg doxophylline, *d*_4_-doxophylline, or *d*_7_-doxophylline). (f, g) Representative images of (f) Masson’s trichrome staining and (g) Picrorius red staining of sham and bleomycin-treated mice (treated or not with 80 mg/kg doxophylline, *d*_4_-doxophylline, or *d*_7_-doxophylline). *p* values: *, *p* < 0.05; **, *p* < 0.01; ***, *p* < 0.001; ****, *p* < 0.0001.

In the second setting of pulmonary damage (Figure 7a), *P. aeruginosa* injection induced increased total BAL cellularity in vehicle-treated and *d*_4_-treated mice compared with sham mice (Figure 7b). Doxophylline and *d*_7_-doxophylline treatment significantly reduced the number of BAL cells (Figure 7b). Lung edema at day 7 was measured as a ratio of wet to dry weight of excised lung tissue (Figure 7c). Administration of doxophylline and *d*_7_-doxophylline considerably decreased *P. aeruginosa*-induced lung inflammation in comparison with vehicle-treated animals and *d*_4_-treated mice. MPO activity showed increased polymorphonuclear cell infiltration in tissues from vehicle-treated mice, whereas treatment with doxophylline and *d*_7_-doxophylline significantly reduced the MPO activity while *d*_4_-doxophylline had a lesser nonsignificant effect (Figure 7d). Histological examination by H&E staining showed a reduction of the inflammatory interstitial infiltrate in the groups treated with doxophylline and its analogues, especially in the doxophylline- and *d*_7_-treated mice compared with the *d*_4_-treated ones (Figure 7e).

**Figure 7:**
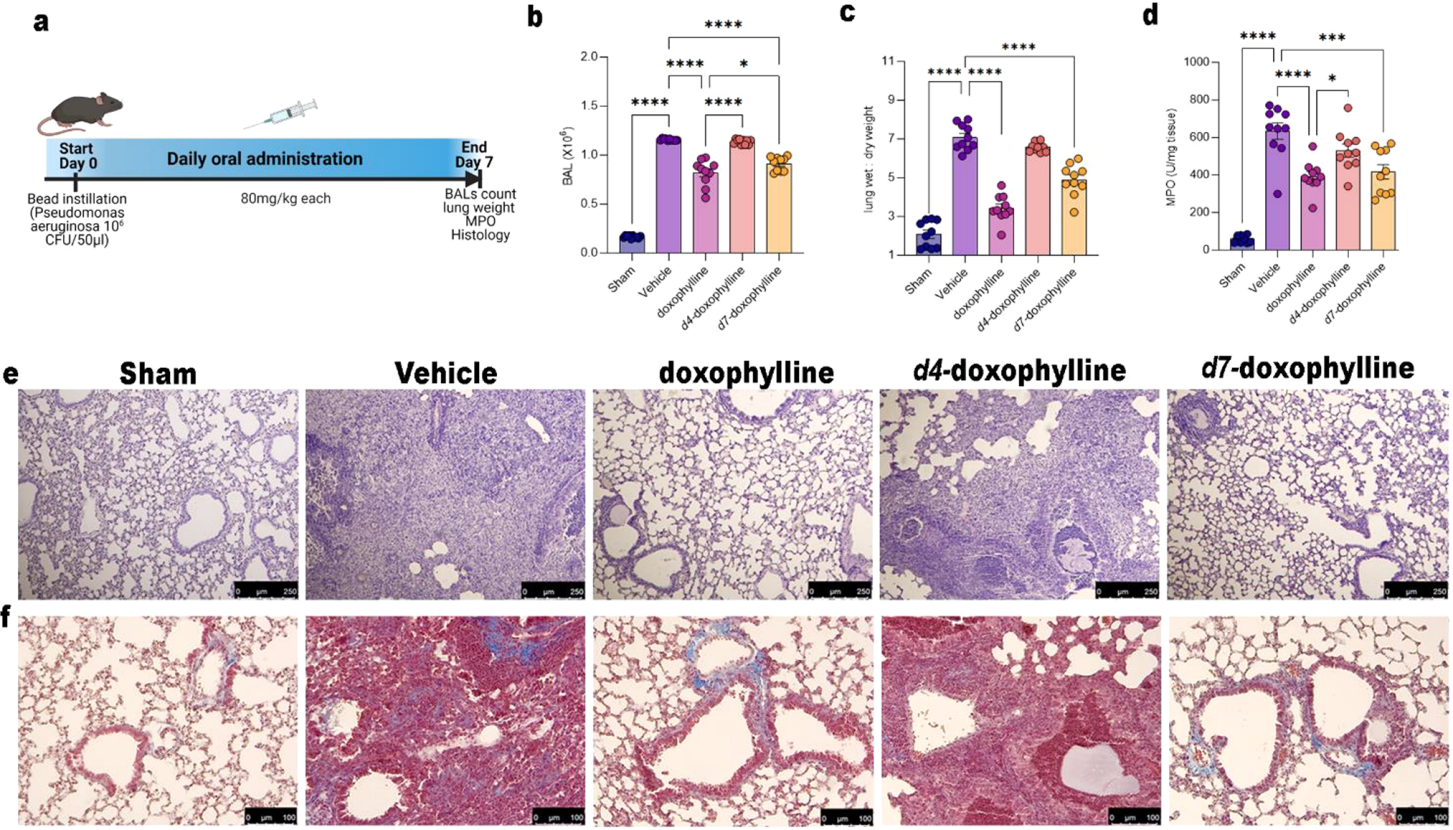
Model of pulmonary fibrosis induced by *P. aeruginosa*. (a) Representative scheme of *P. aeruginosa*-induced lung injury model. (b) Total BAL cellularity of sham and *Pseudomonas*-treated mice (treated or not with 80 mg/kg doxophylline, *d*_4_-doxophylline, or *d*_7_-doxophylline). Results are reported as mean ± SEM of 10 mice for all groups. (c) Wet/dry lung weight ratio of sham and *Pseudomonas*-treated mice (treated or not with 80 mg/kg doxophylline, *d*_4_-doxophylline, or *d*_7_-doxophylline). Results are reported as mean ± SEM of 10 mice for all groups. (d) MPO activity in lungs of sham and *Pseudomonas*-treated mice (treated or not with 80 mg/kg doxophylline, *d*_4_-doxophylline, or *d*_7_-doxophylline). Results are reported as mean ± SEM of 10 mice for all groups. (e) Representative images of H&E staining of sham and *Pseudomonas*-treated mice (treated or not with 80 mg/doxophylline, *d*_4_-doxophylline, or *d*_7_-doxophylline). (f) Representative images of Masson’s trichrome staining of sham and *Pseudomonas*-treated mice (treated or not with 80 mg/doxophylline, *d*_4_-doxophylline and *d*_7_-doxophylline). *p* values: *, *p* < 0.05; **, *p* < 0.01; ***, *p* < 0.001; ****, *p* < 0.0001.

The trend of an effect of doxophylline and *d*_7_-doxophylline and a lesser or no effect from *d*_4_-doxophylline was observed also in other parameters investigated, such as collagen deposition (Figure 7f), immunohistochemistry of nitrotyrosine (Figure S2a), and PAR (Figure S2b). Finally, TUNEL assays were done to evaluate apoptosis in treated tissues, and again there was an increased apoptotic signature in lungs exposed to *P. aeruginosa* that was reverted both by doxophylline and *d*_7_-doxophylline, whereas *d*_4_-doxophylline did not have any effect (Figure S2c).

Overall, the two animal models are concordant in showing that doxophylline has an important protective effect in lung injury and that this is mimicked by *d*_7_-doxophylline, whereas *d*_4_-doxophylline has a lesser effect or no effect, according to the model used. Even if there are no sufficient elements to draw a clear correlation between the metabolic stabilities of *d*_4_- and *d*_7_-doxophylline, the corresponding plasma levels of metabolites, and the *in vivo* efficacy, it is evident that the different deuteration patterns lead to distinct metabolite scenarios, which in turn appears to influence the effect on lung injury. Indeed, it must be taken into account that the pharmacological activity of methylxanthines observed *in vivo* is the result of the interaction of both the drug itself and its structurally similar metabolites with multiple targets that are still not fully elucidated and that on each of these targets every single metabolite has a different potency. It must be also recognized that besides the metabolic fate, other factors might contribute to determining such differences *in vivo*, including an effect of deuterium incorporation on plasma protein binding.^32^

Bronchiectasis is characterized by permanent enlargement of peripheral bronchi accompanied by repeated respiratory infections, disabling productive cough, and shortness of breath, resulting in loss of lung function.^33,34^ While this disorder shows an ever-increasing and worrying prevalence,^35^ no effective pharmacological treatment is currently approved, and the search for a therapy is complicated by the lack of an understanding of its pathophysiology and by the complex etiology of the disease.^36^ Indeed, it can be the result of several underlying causes, including infections by *P. aeruginosa*, *Haemophilus influenzae*, or other pathogens, dysregulated immunity, and impaired mucociliary clearance. Therefore, no animal model closely recapitulates the disorder, and different preclinical settings must be used to represent the different subpopulations of patients.^37^ In spite of the surge of interest in this disease, only a few disease-modifying agents are being evaluated in clinical trials (*e.g.*, A4 leukotriene hydrolases, dipeptidyl peptidase-I inhibitors, elastase inhibitors, and CXC chemokine receptor 2 antagonists),^38^ and they mainly target neutrophiles, the most abundant population recruited in airways, together with macrophages.^34,39^ In this context, doxophylline might represent an effective treatment as it both relaxes airway smooth muscle and has anti-inflammatory properties, with a profound impact not only on neutrophils but also on monocytes and alveolar macrophages. In February 2014, the U.S. Food and Drug Administration granted an orphan drug designation to doxophylline for treatment of bronchiectasis.^40^ Moreover, a couple of patents claim a pharmaceutical composition comprising doxophylline and the mucolytic agent erdosteine to be used in treatment of bronchiectasis^41^ and dosage forms of doxophylline to treat orphan respiratory diseases.^42^ However, no data have been reported to support this hypothesis to date.^7^ In this Letter, we have shown for the first time that both doxophylline and its *d*_7_ analogue are effective in two animal models of chronic lung diseases that partly recapitulate bronchiectasis. Contrary to our expectations, no improvement in pharmacokinetics between doxophylline and its *d*_4_ and *d*_7_ analogues was observed, offering an enlightening example of a deuterium-promoted multidirectional metabolic switch and confirming the challenges associated with precision deuteration in drug R&D.

## Supporting Information Available

Chemistry, determination of purity and HRMS spectra, comparative pharmacokinetic study, and *in vivo* efficacy study (PDF)

## Abbreviations

AUC: area under the curve
BAL: bronchoalveolar lavage
BLM: bleomycin
CL: clearance
CL/F: apparent clearance
CNS: central nervous system
COPD: chronic obstructive pulmonary disease
CYP: cytochrome P450
KIE: kinetic isotope effect
GINA: Global Initiative for Asthma
GOLD: Global Initiative for Chronic Obstructive Lung Disease
H&E: hematoxylin and eosin
HDAC: histone deacetylase
MPO: myeloperoxidase
PAR: proteinase-activated receptor
PDE: phosphodiesterase
PI3K: phosphoinositide 3-kinase
PK: pharmacokinetic
PKC: protein kinase C
R&D: research and development
SEM: standard error of the mean
T-CHO: 7-theophylline acetaldehyde
T-COOH: 7-theophylline acetic acid
TLC: thin-layer chromatography
