# Correction to “An Unexpected Deuterium-Induced Metabolic Switch in Doxophylline”

**DOI:** 10.1021/acsmedchemlett.3c00497

**Published:** 2023-11-27

**Authors:** Silvio Aprile, Giorgia Colombo, Marta Serafini, Rosanna Di Paola, Federica Pisati, Irene Preet Bhela, Salvatore Cuzzocrea, Giorgio Grosa, Tracey Pirali

In the original published version of this article, Figure 6g included an incorrect image. The correct image is as follows:

**Figure 6:**
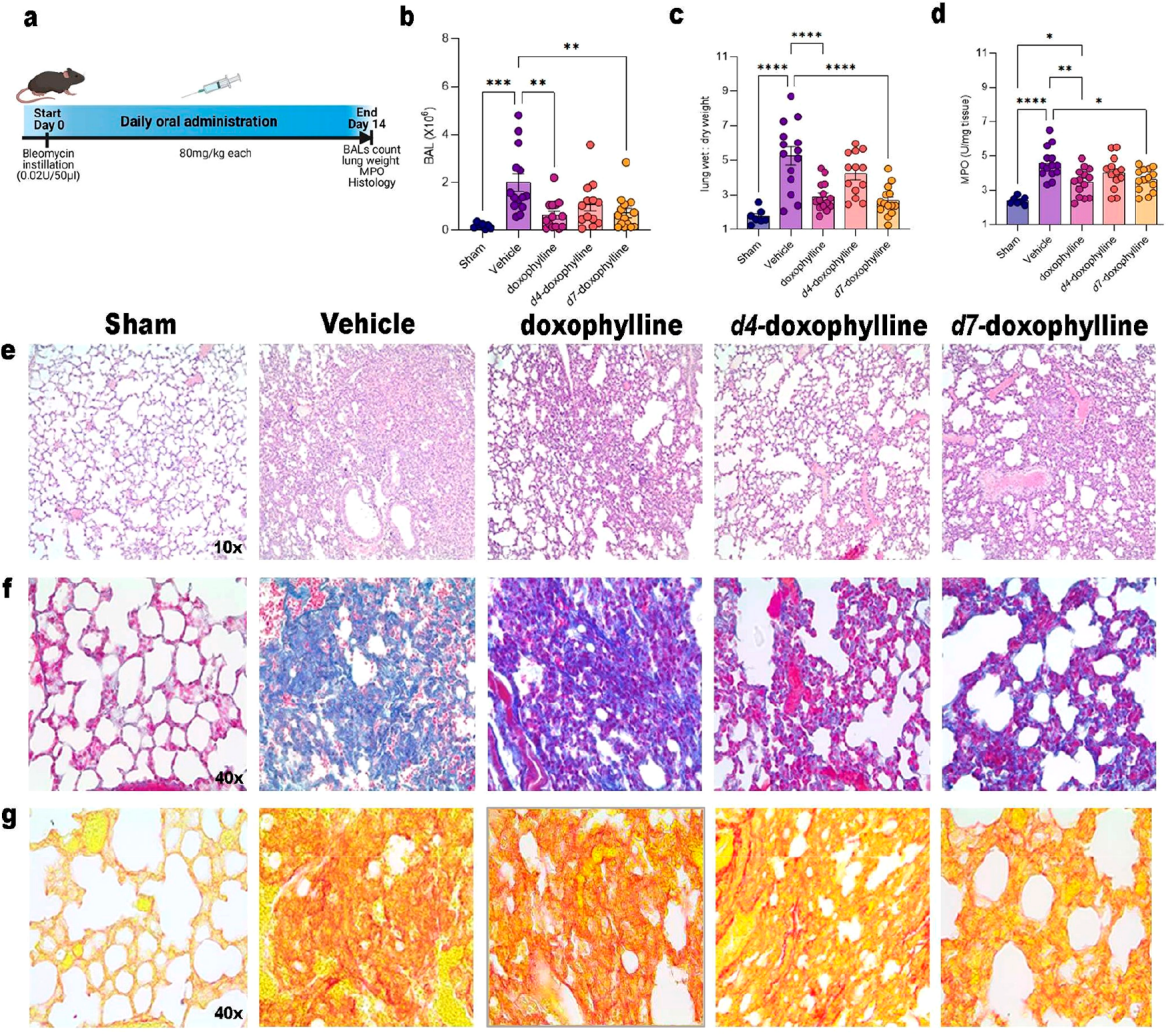
Model of pulmonary fibrosis induced by bleomycin. Doxophylline and *d*_7_-doxophylline attenuate BLM-induced structural damage and lung fibrosis in mice. (a) Representative scheme of BLM-induced lung injury model. (b) Total BAL cellularity of sham (not treated mice) and bleomycin-treated mice (treated or not with 80 mg/kg doxophylline, *d*_4_-doxophylline, or *d*_7_-doxophylline). Mean ± SEM of 3 independent experiments. (c) Wet/dry lung weight ratio of sham and bleomycin-treated mice (treated or not with 80 mg/kg doxophylline, *d*_4_-doxophylline, or *d*_7_-doxophylline). Mean ± SEM of 3 independent experiments. (d) MPO activity in lungs of sham and bleomycin-treated mice (treated or not with 80 mg/kg doxophylline, *d*_4_-doxophylline, or *d*_7_-doxophylline). Mean ± SEM of 3 independent experiments. (e) Representative images of hematoxylin and eosin staining of sham and bleomycin-treated mice (treated or not with 80 mg/kg doxophylline, *d*_4_-doxophylline, or *d*_7_-doxophylline). (f) Representative images of Masson’s trichrome staining and (g) Picrorius red staining of sham and bleomycin-treated mice (treated or not with 80 mg/kg doxophylline, *d*_4_-doxophylline, or *d*_7_-doxophylline). *p* value: * *p* < 0.05; ** *p* < 0.01; *** *p* < 0.001̧**** *p* < 0.0001.

