# Supplementary material for: An Unexpected Deuterium-Induced Metabolic Switch in Doxophylline
Source: ACS Med Chem Lett. 2022 Jul 14;13(8):1278–85. doi: 10.1021/acsmedchemlett.2c00166 (PMC9377007; doi:10.1021/acsmedchemlett.2c00166)
Supplement: Supplementary file 1 — ml2c00166_si_001.pdf [file ml2c00166_si_001.pdf]

SUPPORTING INFORMATION

An unexpected deuterium-induced metabolic switch in doxophylline

Silvio Aprile,[1‡] Giorgia Colombo,[1‡] Marta Serafini,[1‡†] Rosanna Di Paola,[2] Federica Pisati,[3] Irene Preet Bhela,[1] Salvatore Cuzzocrea,[2] Giorgio Grosa,[1] Tracey Pirali[1*]

[1] Department of Pharmaceutical Sciences; Università del Piemonte Orientale; Novara, 28100; Italy, [2] Department of Chemical, Biological, Pharmaceutical and Environmental Sciences; Università di Messina; Messina, 98166; Italy, [3] Histopathology Unit, Cogentech S.C.a.R.L, Milan Italy

Table of contents:

- **p. S2 Chemistry**
- **p. S4 Determination of purity by LC-UV of deuterated doxophyllines and HRMS spectra**
- **p. S7 Comparative pharmacokinetic study**
- **p. S14 In vivo efficacy study**
- **p. S18 References**

- **Chemistry**

General experimental methods

Commercially available reagents and solvents were used as purchased without further purification. When needed, solvents were distilled and stored on 4A molecular sieves. Reactions were monitored by thin layer chromatography (TLC) carried out on 5 cm × 20 cm silica gel plates with a layer thickness of 0.25 mm, using UV light as a visualizing agent. When necessary, TLC plates were visualized with aqueous $KMnO_4$ reagent. Column chromatography was performed on flash silica gel using Kieselgel 60 silica gel (particle size 0.040−0.063 mm, 230−400 mesh). Mps were determined in open glass capillary with a Stuart scientific SMP3 apparatus. All the target compounds were checked by IR (FT-IR Bruker Alpha II), ^{1}H-NMR and ^{13}C-NMR (Bruker Avance Neo 400 MHz), HRMS (Thermo Fisher Q-Exactive Plus) equipped with an Orbitrap (ion trap) mass analyzer. Chemical shifts are reported in parts per million (ppm). No unexpected or unusually high safety hazards were encountered.

7-Theophylline acetic acid (T-COOH), 7-hydroxyethyl theophylline (Eto), and theophylline were purchased from Merck Life Science S.r.l. Milan, Italy)

*Synthesis of d_4-2-(bromomethyl)-1,3-dioxolane (**15**)*

Acetaldehyde (500 mg, 11.35 mmol) is solubilized in CH_2Cl_2 (1 mL) under nitrogen atmosphere and the reaction is cooled at 0 °C. Then, bromine (1.81 g, 11.35 mmol) is added and the reaction is stirred at 0 °C for additional 10 minutes and at room temperature for 2 hours, affording the 2-bromoacetaldehyde **13**. The solution of 2-bromoacetaldehyde **13** is slowly added to a solution of d_4-ethylene glycol (748 mg, 11.35 mmol) and (±)-camphor-10-sulfonic acid (262 mg, 1.13 mmol) in toluene (5 mL) under nitrogen atmosphere. The mixture is stirred at 78 °C overnight. Then, saturated aqueous solution of $Na_2S_2O_3$ is added and the aqueous phase is extracted with CH_2Cl_2 (x2). The organic phases are dried over sodium sulfate and evaporated. The crude material is purified by column chromatography using PE/CH_2Cl_2 95:5, PE/CH_2Cl_2 8:2 and PE/CH_2Cl_2 7:3 as eluents, yielding compound **15** (1.81 g, 10.56 mmol, 93%) as a colorless oil.

^{1}H-NMR (400 MHz, $CDCl_3$): δ 5.14 (t, J = 3.5 Hz, 1H), 3.40 (d, J = 3.6 Hz, 2H).

*Synthesis of 7-((1,3-dioxolan-2-yl)methyl-d_4)-1,3-dimethyl-1H-purine-2,6(3H,7H)-dione, d_4-doxophylline (d4-doxophylline **16**)*

To a solution of 1,3-dimethyl-1*H*-purine-2,6(3*H*,7*H*)-dione **1** (100 mg, 0.56 mmol) in DMF (2 mL), potassium carbonate (93 mg, 0.67 mmol) and d_4-2-(bromomethyl)-1,3-dioxolane 10 (105 mg, 0.62 mmol) are added in order. The reaction is stirred at 115 °C for 16 hours, then water is added and the aqueous phase is extracted with EtOAc (x7). The organic phases are dried over sodium sulfate and the volatile are removed under vacuo. The crude material is purified by column chromatography using PE/EtOAc 4:6, PE/EtOAc 2:8 and EtOAc as eluents, yielded compound **16** (124 mg, 0.46 mmol, 82%) as a white solid.

^{1}H-NMR (400 MHz, DMSO-d_6): δ 8.01 (s, 1H), 5.20 (t, J = 4.2 Hz, 1H), 4.42 (d, J = 4.2 Hz, 2H), 3.42 (s, 3H), 3.22 (s, 3H). ^{13}C-NMR (400 MHz, DMSO-d_6): δ 154.9, 151.4, 148.5, 143.6, 106.7, 101.1, 64.2, 48.1, 29.9,

28.0. MS: M+1 271. HRMS (ESI) m/z $(M+H)^+$ calcd for $C_{11}H_{10}D_4N_4O_4$ 271.1339, found 271.1333. IR (neat): ṽ = 3109, 2952, 1694, 1651, 1544, 1182, 1020, 751, 617, 417 cm^{-1}. Melting point: 148.0 – 148.9 °C, dec.

*Synthesis of d_7-2-(bromomethyl)-1,3-dioxolane (**19**)*

d_4-Acetaldehyde (500 mg, 10.40 mmol) is solubilized in CH_2Cl_2 (1 mL) under nitrogen atmosphere and the reaction is cooled at 0 °C. Then, bromine (1.66 g, 10.40 mmol) is added and the reaction is stirred at 0 °C for additional 10 minutes and at room temperature for 2 hours, affording the *d_3*-2-bromoacetaldehyde **12**. The solution of *d_3*-2-bromoacetaldehyde **18** is slowly added to a solution of *d_4*-ethylene glycol (687 mg, 10.40 mmol) and (±)-camphor-10-sulfonic acid (241 mg, 1.04 mmol) in toluene (5 mL) under nitrogen atmosphere. The mixture is stirred at 78 °C overnight. Then, saturated aqueous solution of $Na_2S_2O_3$ is added and the aqueous phase is extracted with CH_2Cl_2 (x2). The organic phases are dried over sodium sulfate and evaporated. The crude material is purified by column chromatography using PE/CH_2Cl_2 95:5, PE/CH_2Cl_2 8:2 and PE/CH_2Cl_2 7:3 as eluents, yielding compound **19** (1.52 g, 8.74 mmol, 84%) as a colorless oil.

*Synthesis of 7-((1,3-dioxolan-2-yl)methyl-d_7)-1,3-dimethyl-1H-purine-2,6(3H,7H)-dione, d_7-doxophylline (d7-doxophylline **20**)*

To a solution of 1,3-dimethyl-1*H*-purine-2,6(3*H*,7*H*)-dione **1** (200 mg, 1.11 mmol) in DMF (4 mL), potassium carbonate (184 mg, 1.33 mmol) and *d_7*-2-(bromomethyl)-1,3-dioxolane 12 (212 mg, 1.22 mmol) are added in order. The reaction is stirred at 115 °C for 16 hours, then water is added and the aqueous phase is extracted with EtOAc (x7). The organic phases are dried over sodium sulfate and the volatile are removed under vacuo. The crude material is purified by column chromatography using PE/EtOAc 4:6, PE/EtOAc 2:8 and EtOAc as eluents, yielded compound **20** (240 mg, 0.88 mmol, 79%) as a white solid.

^{1}H-NMR (400 MHz, DMSO-*d_6*): δ 8.01 (s, 1H), 3.42 (s, 3H), 3.22 (s, 3H). ^{13}C-NMR (400 MHz, DMSO-*d_6*): δ 155.0, 151.4, 148.5, 143.5, 106.7, 101.0, 64.2, 48.0, 29.9, 28.0. MS: M+1 274. HRMS (ESI) m/z $(M+H)^+$ calcd for $C_{11}H_7D_7N_4O_4$ 274.1527, found 274.1519. IR (neat): ṽ = 3109, 2953, 1694, 1650, 1543, 1204, 1024, 751, 612, 417 cm^{-1}. Melting point: 150.7 – 151.5 °C, dec.

- **Determination of purity by LC-UV of deuterated doxophyllines and HRMS spectra**

- Instrumentation: Shimadzu HPLC system LC-10AD series
- Column: Phenomenex Sinergy Polar 150 × 4.6 mm (4 µm *d.p.*)
- Eluants: solvent A= 0.2% formic acid aqueous solution, solvent B= 0.2% formic acid in acetonitrile
- Flow rate: 1 mL/min
- Injection volume: 20 µL
- Wavelength: 272 nm
- gradient elution program 0.0 min B=10%, 5.0 min B=25%, 10.0 min B=80%, 14.50 min B=80%, 15.0 min B=10, 20.0 min B=10

***d4*-doxophylline** purity HPLC >95%

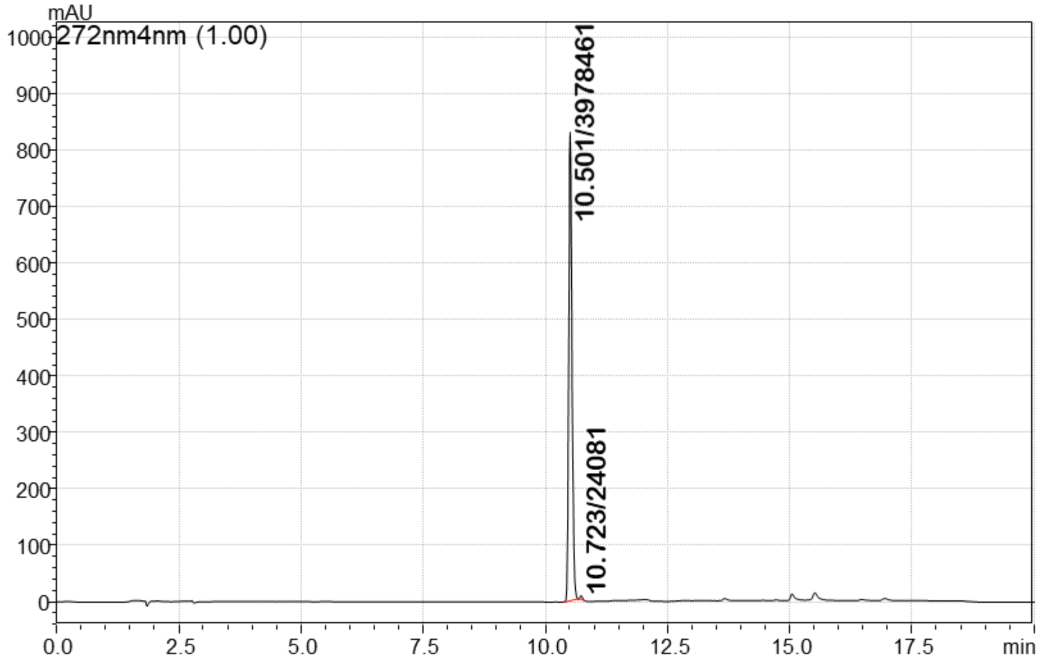

***d7*-doxophylline** purity HPLC >95%

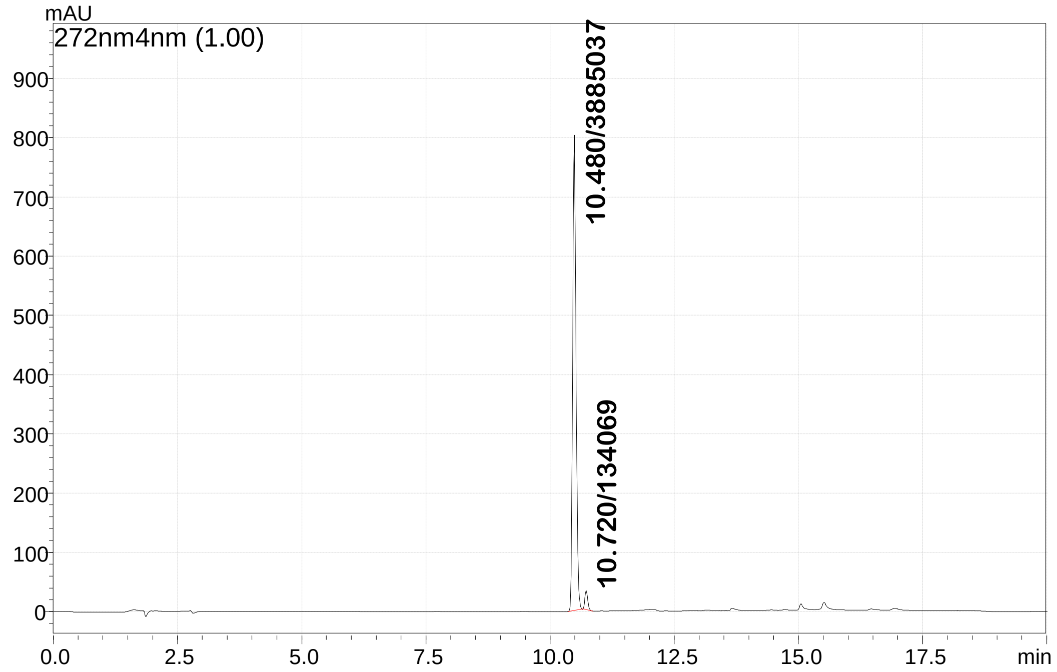

HRMS spectra of *d4*- and *d7*-doxophylline

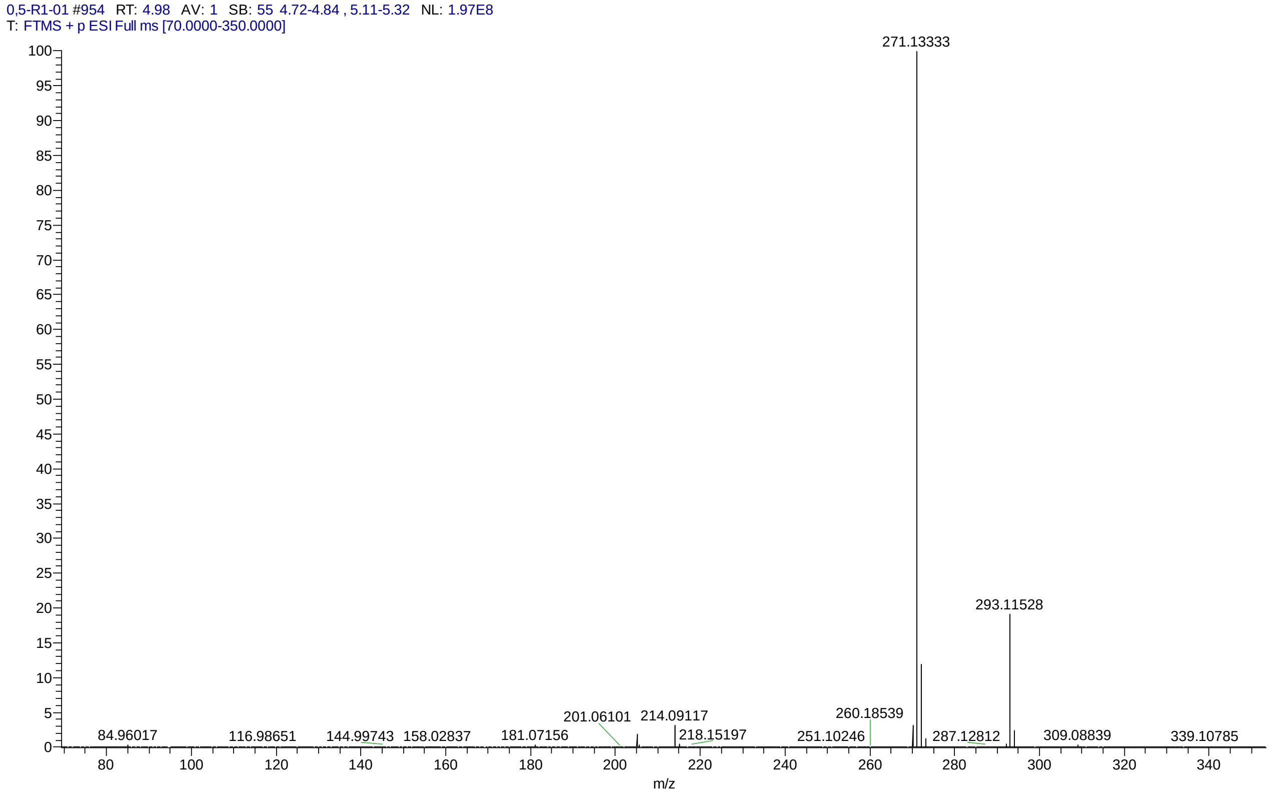

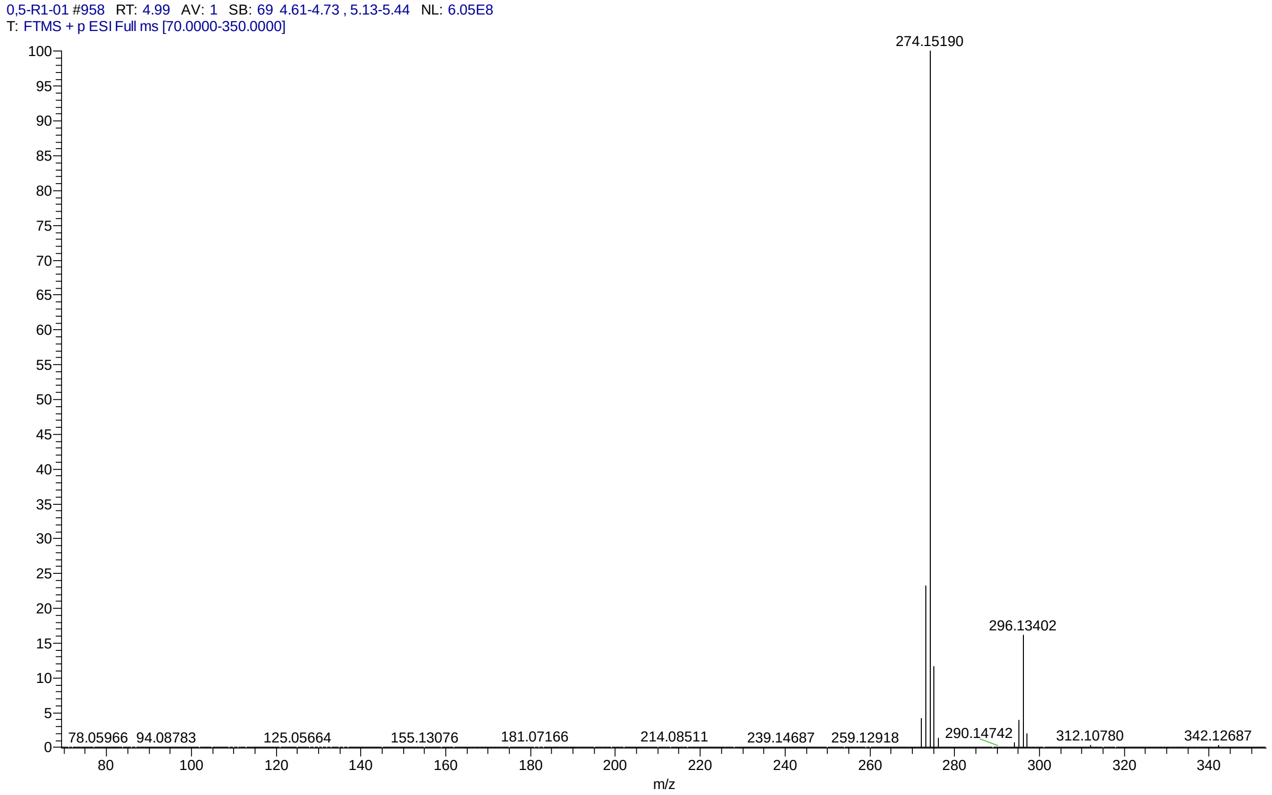

- ## Comparative pharmacokinetic study

Animals.

A total of 96 CD1 male mice, approximately 6 weeks old and with a body weight of approximately 30 g, were purchased from Envigo RMS srl, (San Pietro al Natisone, UD, Italy). Four males Göttingen minipigs approximately 4-5 months old and weighing 9-11 Kg were purchased from Ellegaard Göttingen Minipigs (Dalmose, Denmark).

Protocol for drug administration and blood sampling.

Mice.

Blood samples were collected under light isoflurane anaesthesia from the sublingual vein at the following time points (n=3): pre-dose and 5, 15, 30 minutes, 1, 2, 4, 8 and 24 hours after i.v. dosing (20 mg/Kg, dose volume 5 mL/Kg) and pre-dose and 15, 30 minutes, 1, 2, 4, 8 and 24 hours after oral gavage dosing (80 mg/Kg, dose volume 5 mL/Kg). Each animal was sampled on each day at a maximum of two alternating time points. Samples were transferred into tubes containing K_3EDTA as anticoagulant, centrifuged, and stored at -80 °C.

Minipig.

Blood samples were collected from the jugular vein at the following time points (n=4): pre-dose and 5, 15, 30 minutes, 1, 2, 4, 8 and 24 hours after i.v. dosing (5 mg/Kg, dose volume 1 mL/Kg) and pre-dose and 15, 30 minutes, 1, 2, 4, 8 and 24 hours after oral gavage dosing (20 mg/Kg, dose volume 4 mL/Kg). Samples were transferred into tubes containing K_3EDTA as anticoagulant, centrifuged, and stored at -80 °C.

Sample preparation for HPLC-MS analysis.

A volume of 50 µL of caffeine solution (1 mg/L in water-methanol 1:1) were added to plasma sample aliquots (50 µL); after the addition of the precipitant solution (100 µL of 10% ice-cold trichloroacetic acid in water), samples were centrifuged at 13,000 rpm for ten minutes and the supernatant injected onto the HPLC system (see supporting information for chromatographic conditions). If the levels of analytes were over range, plasma samples were preventively diluted with blank plasma.

Data analysis.

Quantification of doxophylline and its deuterated analogues *d4* and *d7* as well as their main metabolites, 7-theophylline acetic acid (T-COOH), 7-hydroxyethyl theophylline (Eto) and theophylline, was performed by using the calibration curves obtained with the corresponding working standards of the analytes and using caffeine as internal standard.

The pharmacokinetic parameters for doxophylline and its deuterated analogues were calculated by plotting the plasma concentration curves versus time obtained from the average values of three animals, using PK Solver 2.0.[1]

HPLC-HRMS analysis

	Hybrid quadrupole-orbitrap, Thermo Scientific Q-exactive *Plus*, equipped with a Vanquish UHPLC system.
Column:	Kinetex C18 (150 × 2 mm, 2.6 µm d_p) (Phenomenex).
Mobile Phase	Phase A: 0.1% formic acid in water UHPLC grade.<br>Phase B: 0.1% formic acid in methanol UHPLC grade.
Analysis mode:	Gradient of concentration.

Time (min)	% B
0.00	10
2.80	20
5.40	70
5.50	95
9.00	95
9.20	10
13.00	10

Flow rate:	0.270 mL/min
Column temperature:	40 °C
Sample temperature:	15 °C
Injected volume:	5 µL

The operating conditions of the mass spectrometer were as follows: positive mode; sheath gas flow rate, 45 Auxiliary Units (A.U.), auxiliary gas flow rate, 10 A.U.; sweep gas flow rate, 2 A.U.; spray voltage, 3.50 kV; capillary temperature, 300 °C; auxiliary gas heater temperature, 350 °C.

Data were acquired in full-scan/parallel reaction monitoring (PRM) modes using a mass scan range *m/z* 70-350. The collision energy was set at 33-36 % levels. The ions monitored were reported in the following tables.

Figure S1. Chromatogram depicting all the analytes monitored

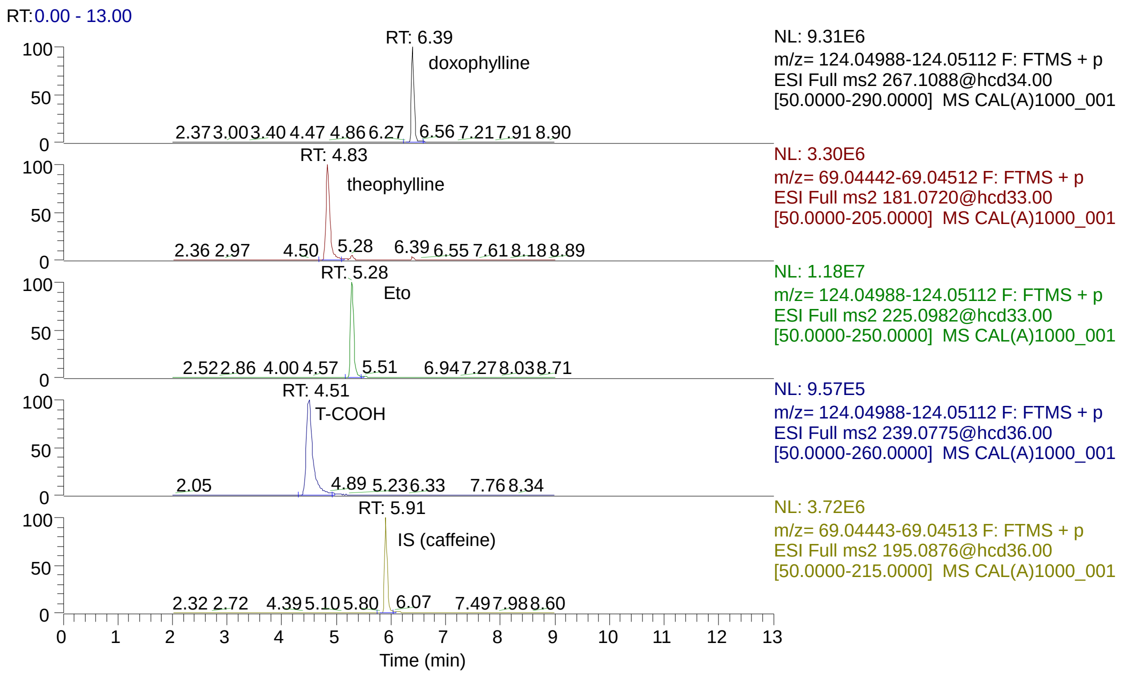

Table S1. Ions monitored in mice and minipig plasma samples treated with doxophylline.

	Molecula formula	$[M+H]^+$	Structure
**Doxophylline**	$C_{11}H_{14}N_4O_4$	267.1088	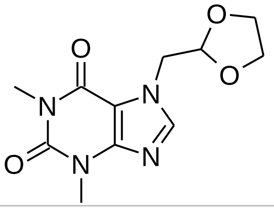
**T-COOH**	$C_9H_{10}N_4O_4$	239.0775	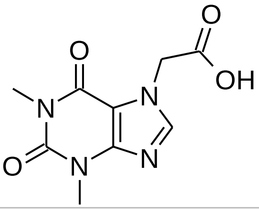
**Etophylline**	$C_9H_{12}N_4O_3$	225.0982	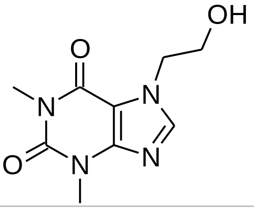
**Theophylline**	$C_7H_8N_4O_2$	181.0720	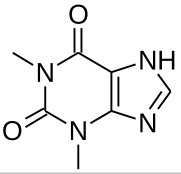
**Dm-doxophylline**	$C_{10}H_{12}N_4O_4$	253.0931	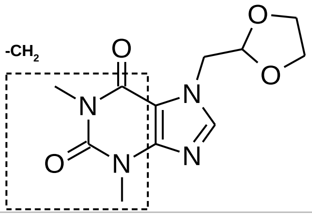
**Dh-doxophylline**	$C_{11}H_{12}N_4O_4$	265.0931	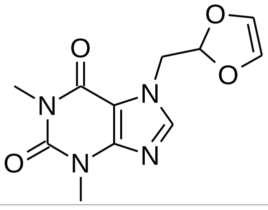

Table S2. Ions monitored in mice and minipig plasma samples treated with *d4*-doxophylline.

	Molecula formula	$[M+H]^+$	Structure
d4	$C_{11}H_{10}D_4N_4O_4$	271.1339	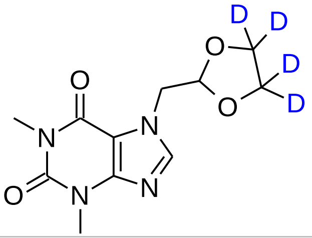
**T-COOH**	$C_9H_{10}N_4O_4$	239.0775	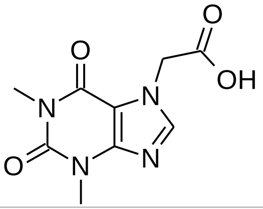
**Etophylline**	$C_9H_{12}N_4O_3$	225.0982	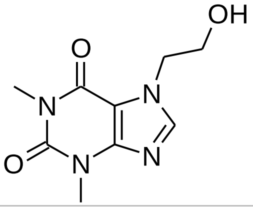
**Theophylline**	$C_7H_8N_4O_2$	181.0720	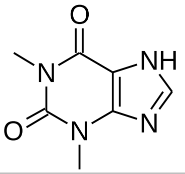
***d4*-dm**	$C_{10}H_8D_4N_4O_4$	257.1182	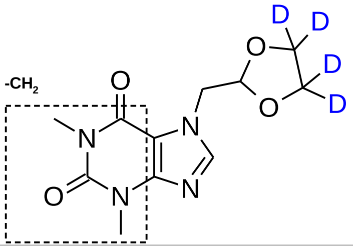
***d4*-dh**	$C_{11}H_{10}D_2N_4O_4$	267.1057	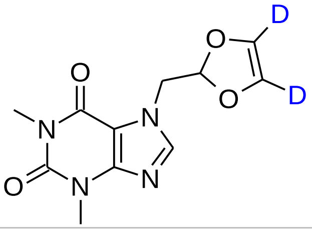

Table S3. Ions monitored in mice and minipig plasma samples treated with *d7*-doxophylline.

	**Molecula formula**	**[M+H]$^+$**	**Structure**
d7	$C_{11}H_7D_7N_4O_4$	274.1527	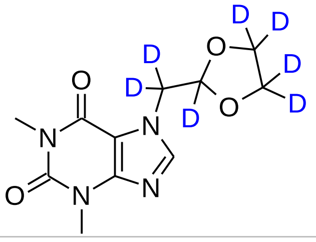
***d7*-COOH**	$C_9H_8D_2N_4O_4$	241.0900	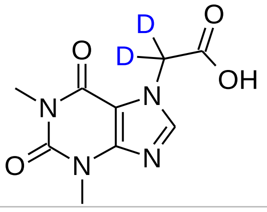
***d7*-Etophylline**	$C_9H_9D_3N_4O_3$	228.1170	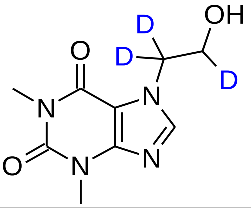
**Theo**	$C_7H_8N_4O_2$	181.0720	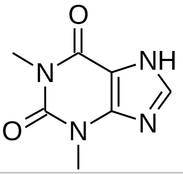
***d7*-dm**	$C_{10}H_5D_7N_4O_4$	260.1371	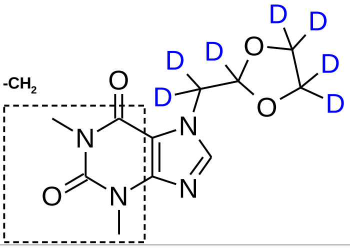
***d7*-dh**	$C_{11}H_7D_5N_4O_4$	270.1245	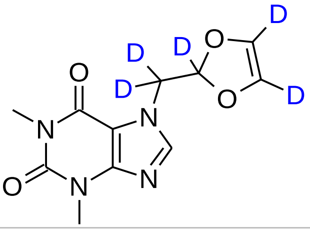

HRMS^2 characterization of doxophyllines metabolites which the reference standards were not available

Dm-doxophylline

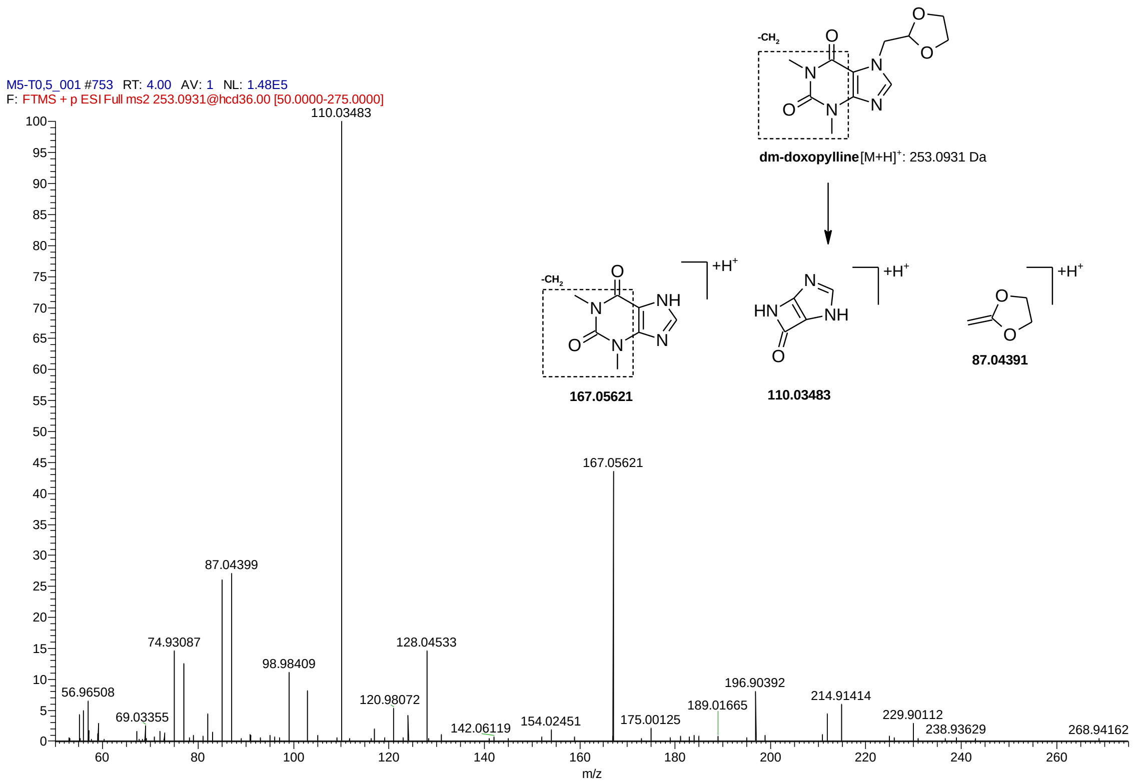

Dh-doxophylline

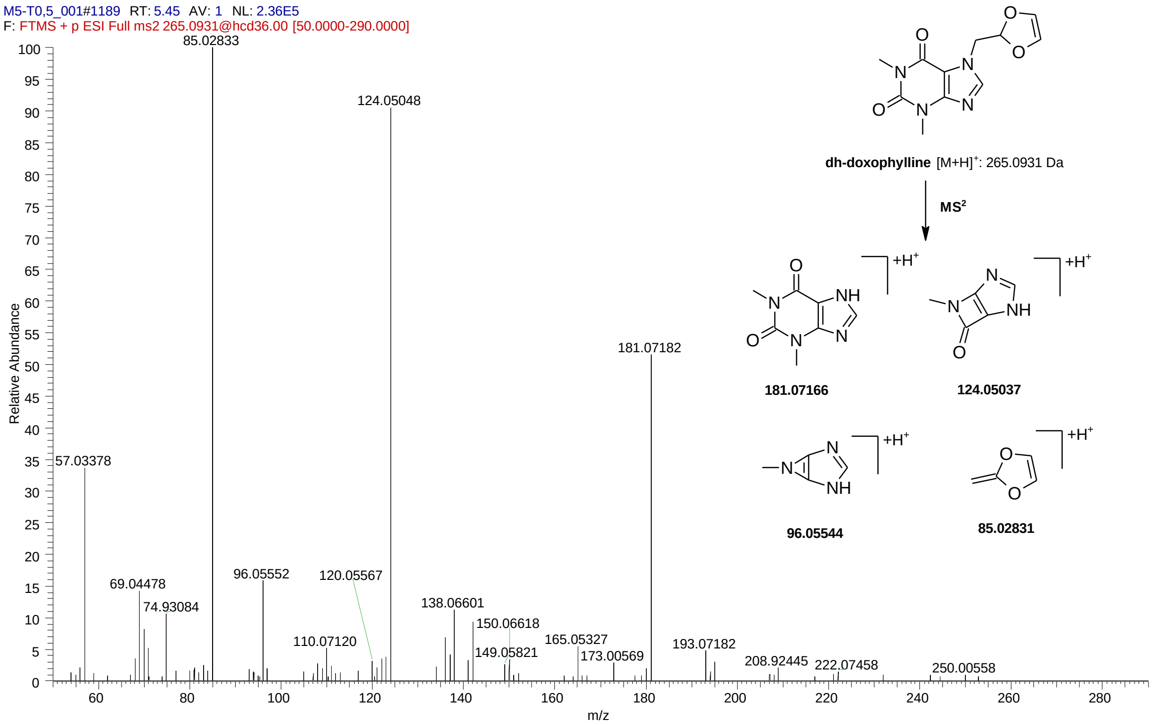

- ***In vivo* efficacy study**

Bleomycin model

Induction.

8-week-old male C57Bl/6 mice (Envigo, Indianapolis, USA) weighing between 25 and 30 g were used. Animal care was in compliance with Italian regulations on protection of animals used for experimental purposes and were authorized by the Ministry of Health. We administered BLM (0.02U/50µl) by mouse pharyngeal aspiration, under isoflurane anaesthesia.[2] Doxophylline, *d4* or *d7* (80 µg/kg, solubilized in 0.9% NaCl solution) or saline treatment (vehicle) was given orally (gavage administration) 1 h before BLM treatment, and then once a day thereafter. Mice were euthanized on day 21 following BLM treatment.

BAL analysis.

BAL was performed through a tracheal cannula using 1 mL aliquots of ice-cold Ca^{2+}/Mg^{2+}-free phosphate-buffered medium (145 mM NaCl, 5 mM KCl, 1.9 mM NaH_2PO_4, 9.35 mM Na_2HPO_4, and 5.5 mM dextrose, pH 7.4). BAL samples were centrifuged at 500×g for 5 min at 4 °C, and cell pellets were washed and resuspended in phosphate-buffered medium. BAL cells were enumerated by counting on a Hemocytometer the presence of the trypan blue stain.

MPO measurement.

Myeloperoxidase (MPO) activity, a marker of polymorphonuclear leukocyte (PMN) infiltration, was determined using MPO Colorimetric Activity Assay Kit (Merck Life Science), according to the manufacturer's protocol. MPO activity was described as the amount of enzyme degrading 1 µM of peroxide/minute at 37°C and was expressed in U/mg of wet tissue.

Evaluation of fluid content in lungs.

The wet lung weight was calculated after harvesting the lungs, without any extraneous tissue. The lungs were subjected for 72 hours at 100°C and the dry weight was measured. Water content was calculated by subtracting dry weight from wet weight.

Histology.

Lungs were fixed with 10% buffered formalin at room temperature for 48h, dehydrated in 70% ethanol, and embedded in paraffin with Diapath automatic processor. Sections (4 µm thick) were stained with haematoxylin and eosin (H&E) according to standard protocol and samples were mounted in Eukitt (Bio-Optica). To visualize stromal fibres in lung sections Picrosirius Red staining (Scy Tek Lab, SRS-IFU) and Masson's Trichrome (Diapath, 010210) were performed.

Pseudomonas aeruginosa model

Animals.

C57BL-6 mice, aged 12–16 weeks old, were used (obtained from Envigo Laboratories) placed in cages with rodent chow and water. Cages were placed in controlled room at 22±1 °C with a 12 hours dark and light cycle. The entire study was permitted by the University of Messina Review Board for animals' care. In vivo

experiments were performed following the new regulations of USA (Animal Welfare Assurance No A5594-01), Europe (EU Directive 2010/63), Italy (D.Lgs 2014/26) and the ARRIVE guidelines.

PA-laden agar beads.

Tryptic soya agar beads laden with PA were produced as described. The mucoid PA strain NH57388A, was utilized. NH57388A-laden agar beads were prepared the day before inoculation, stored overnight at 4 °C, suspended in sterile PBS to deliver approximately 1 Å~ 106 CFU in 50 μ l per mouse. The inoculation dose was chosen based on previous studies demonstrating high acute mortality with higher inoculation doses.

Trans-tracheal instillation of beads.

Mice were anaesthetized with inhaled isoflurane delivered initially in an anaesthetic box until unconscious, transferred to a surgical board and maintained under anesthesia with isoflurane via nose cone. The ventral cervical region was sterilized and 1-cm midline skin incision made just cranial to the thoracic inlet. The trachea was visualized by blunt dissection. Traction on the lower jaw was used to extend and straighten the trachea and a 22G intravenous catheter (BD Biosciences) used to cannulate the trachea. A 1-ml syringe, pre-filled with beads suspended in PBS, was attached to the cannuale. The cannulae and syringe were left in-situ for 5-seconds following depression of the plunger, followed by slow withdrawal. The animal was elevated head up to 45-degrees. A rapid rise in respiratory rate was frequently observed at this point suggesting accurate intrapulmonary bead instillation. If evidence of respiratory distress (rapid or labored respiration/ gasping) occurred during the procedure anesthesia was rapidly reversed and oxygen alone administered via nose cone. The incision was closed with surgical staples. Buprenorphine analgesia was administered subcutaneously post-operatively. Animals were weighed and scored clinically daily. Experiments were terminated at 2-weeks post-inoculation via anesthetic overdose.

Experimental groups.

Mice were randomly allocated into the following groups. PSEUDOMONAS group: mice were subjected to pseudomonas-induced lung injury (n = 10); PSEUDOMONAS group + doxophylline: Identical to the pseudomonas + vehicle group but were treated daily with doxophylline xo (80 mg/kg os) 1 h after pseudomonas instillation and continued daily for 7 days (n = 10); PSEUDOMONAS group + *d4*- doxophylline: Identical to the pseudomonas + vehicle group but were treated daily with doxophylline deuterated *d4* (80 mg/kg os) 1 h after pseudomonas instillation and continued daily for 7 days (n = 10); PSEUDOMONAS group + *d7*- doxophylline: Identical to the pseudomonas + vehicle group but were treated daily with doxophylline deuterated *d7* (80 mg/kg os) 1 h after pseudomonas instillation and continued daily for 7 days (n = 10); SHAM group: mice will receive the vehicle administration daily for 7 days (n=15); SHAM + doxophylline group: mice received the doxophylline administration daily for 7 (n=10); SHAM + doxophylline deuterated *d4* group: mice received the deudoxophylline administration daily for 7 (n=10); SHAM + doxophylline deuterated *d7* group: mice received the deudoxophylline administration daily for 7 (n=10)

Survival rate.

Mortality was assessed daily up to day 7. On day 7 after pseudomonas administration, surviving mice were sacrificed followed by BAL, MPO, lung edema, collagen determination, histopathology and immunohistochemical localization of nitrotyrosine, PAR, and TUNEL assay.

Bronchoalveolar lavage.

7 days after pseudomonas instillation, mice were euthanized, terminal plasma samples were taken, and the tracheas were cannulated with an I.V. polyethylene catheter (Neo Delta Ven 2, delta Med, Viadana, Italy) equipped with a 24-gauge needle on a 1 mL syringe. Lungs were washed once with 0.5 mL Dulbecco's phosphate-buffered saline (PBS) (GIBCO, Paisley, U.K.). In more than 95% of the mice, the recovery volume was over 0.4 mL. The BAL fluid was spun, the pelleted cells were collected and the supernatants stored at -20 °C. In the presence of trypan blue total BAL cells were counted using a hemocytometer.

Measurement of lung edema.

After 7 days injection of pseudomonas wet lung weights were measured by careful excision of the lung from adjacent tissues. The lungs were dried for 48 h at 180 °C and then weighed. Water content of the lungs was subsequently calculated as the ratio of wet: dry weight of the tissue.

Histological examination.

After 7 days injection of pseudomonas lung biopsies were taken. After fixing the tissues at room temperature in buffered formaldehyde solution (10% in PBS), histological sections were stained by haematoxylin and eosin and evaluated using an Axiovision Zeiss (Milan, Italy) microscope. Randomly chosen sections were examined, 5 fields per sample at a magnification of x100, and the severity of lung fibrosis was scored by blinded observer.

MPO assay.

After 7 days injection of *Pseudomonas aeruginosa*, MPO activity was determined. The lung were removed, weighed, homogenized in a solution containing 0.5% hexadecyltrimethylammonium bromide dissolved in 10 mM potassium phosphate buffer (pH 7) and centrifuged for 30 min at 20,000 x *g* (at 4 °C). The supernatant was then reacted with a solution of 0.1 mM H_2O_2 and tetramethylbenzidine (1.6 mM). The change in absorbance was measured spectrophotometrically at 650 nm. MPO activity was defined as the quantity of enzyme degrading 1 μmol of hydrogen peroxide per min at 37 °C and was expressed in units per gram wet tissue weight.

Immunohistochemical localization of nitrotyrosine, PAR, and TGF-β.

Tyrosine nitration, an index of the nitrosylation of proteins by peroxynitrite and/or ROS, will be determined by immunohistochemistry analysis. At the end of the experiment, the tissues will be fixed in 10% (*w/v*) PBS-buffered formaldehyde, and 8-μm sections were prepared from paraffin- embedded tissues. After deparaffinization, endogenous peroxidase will be quenched with 0.3% hydrogen peroxide (*v/v*) in 60% (*v/v*) methanol for 30 min. The sections will be permeabilized with 0.1% (*w/v*) Triton X-100 in PBS for 20 min. Nonspecific adsorption will be minimized by incubating the section in 2% (*v/v*) normal goat serum in PBS for 20 min. Endogenous biotin or avidin binding sites will be blocked by sequential incubation for 15 min with biotin and avidin (DBA, Milan, Italy), respectively. Sections will be incubated overnight with anti-nitrotyrosine rabbit polyclonal antibody (1:500 in PBS, *v/v*) or anti-poly(ADP-ribose) goat polyclonal antibody

(1:500 in PBS, *v/v*). Sections will be washed with PBS and incubated with appropriate secondary antibodies. Specific labeling will be detected with biotin-conjugated IgG and avidin-biotin peroxidase complex (DBA). To confirm that the immunoreaction for the nitrotyrosine was specific, some sections will be also incubated with the primary antibody (anti-nitrotyrosine) in the presence of excess nitrotyrosine (10 mM) to verify the binding specificity. To verify the binding specificity for PAR, some sections will be also incubated with only the primary antibody (no secondary) or with only the secondary antibody (no primary). In these situations, no positive staining was found in the sections, indicating that the immunoreaction was positive in all the experiments carried out.

TUNEL assay.

TUNEL assay will be conducted by using a TUNEL detection kit according to the manufacturer's instructions (Apotag, horseradish peroxidase kit; DBA). Briefly, sections were incubated with 15 μg/ml proteinase K for 15 min at room temperature and then washed with PBS. Endogenous peroxidase was inactivated by 3% hydrogen peroxide for 5 min at room temperature and then washed with PBS. Sections will be immersed in terminal deoxynucleotidyl transferase buffer containing deoxynucleotidyl transferase and biotinylated dUTP in terminal deoxynucleotidyl transferase buffer, incubated in a humid atmosphere at 37°C for 90 min, and then washed with PBS. The sections will be incubated at room temperature for 30 min with anti- horseradish peroxidase-conjugated antibody, and the signals were visualized with diaminobenzidine.

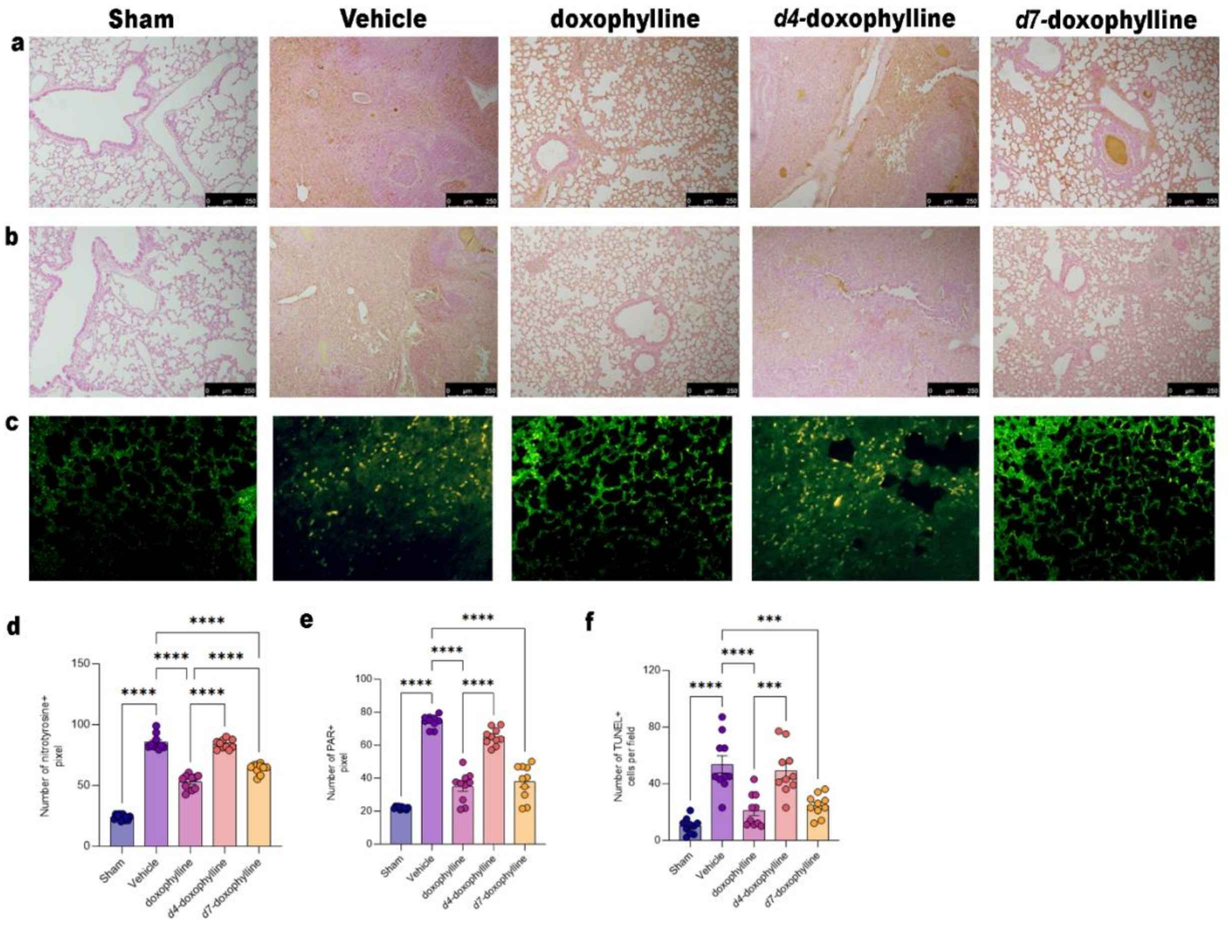

Figure S2: (**a**) Representative images of immunolocalization of nitrotyrosine in lung of sham and Pseudomonas-treated mice (treated or not with 80 mg/kg doxophylline, *d4* and *d7*). Mean ± S.E.M. of 10 mice for all group. (**b**) Representative images of immunolocalization of PAR in lung of sham and Pseudomonas-treated mice (treated or not with 80 mg/kg doxophylline, *d4* and *d7*). Mean ± S.E.M. of 10 mice for all group. (**c**) Effects of doxophylline, *d4* and *d7* analogues on apoptosis *Pseudomonas aeruginosa*-induced mice using TUNEL assay. (**d**) Densitometric analysis of nitrotyrosine positive pixels. (**e**) Densitometric analysis of PAR positive pixels. (**f**) densitometric analysis TUNEL positive cells. Values are means ± SEM of 10 mice for all group.

P value: * p<0.05; ** p<0.01; *** p<0.001¸**** p<0.0001.

- ## References

1. Zhang, Y.; Huo, M.; Zhou, J.; Xie, S., PKSolver: An add-in program for pharmacokinetic and pharmacodynamic data analysis in Microsoft Excel. *Computer methods and programs in biomedicine* **2010,** *99* (3), 306-14.

2. Stevens, L. E.; Arnal-Estapé, A.; Nguyen, D. X., Pre-Conditioning the Airways of Mice with Bleomycin Increases the Efficiency of Orthotopic Lung Cancer Cell Engraftment. *Journal of visualized experiments : JoVE* **2018,** (136).
